# The variability of bacterial communities in both the endosphere and ectosphere of different niches in Chinese chives (*Allium tuberosum*)

**DOI:** 10.1371/journal.pone.0227671

**Published:** 2020-01-16

**Authors:** Yuxin Wang, Chaonan Wang, Yizhu Gu, Pingzhi Wang, Weitang Song, Jinhai Ma, Xiaofei Yang

**Affiliations:** 1 College of Water Resources & Civil Engineering, China Agricultural University, Haidian, Beijing, China; 2 Henan Jiuxing Institute of Biotechnology, Pingdingshan, Henan, China; Free University of Bozen-Bolzano, ITALY

## Abstract

Deciphering the various types of interactions between plants and their microbiomes is a hot topic for research in ecology as well as in plant sciences and agronomy. To analyse and compare the differences in microbial communities in different compartments of Chinese chives, high-throughput sequencing technology was employed to amplify and sequence the V5-V6 region of the 16S rDNA of microorganisms in the leaves, phylloplanes, stems, roots and rhizospheres of Chinese chives. The sequences were clustered by operational taxonomic units (OTUs), and the community composition of bacteria between the endosphere (inner tissues) and ectosphere (outer surfaces) of Chinese chives was analysed based on the OTU. Overall, the results indicated that the endophytic bacteria in Chinese chives mainly include *Proteobacteria*, *Actinobacteria*, and *Actinomycetes*. Alpha diversity index analysis and OTU number analysis showed that the bacterial diversity and richness of the underground plant compartments were higher than those of the above-ground parts. PCoA based on the OTU level showed that the vertical stratification structure of plants and compartments had significant effects on the bacterial community structure. The richness of endophytic bacteria also varied greatly among the different varieties of Chinese chive. A considerable number of endophytic bacteria form symbiotic and mutually beneficial relationships with host plants, which play an important role in regulating host growth, metabolism and stress resistance. Further investigations are needed to uncover the evolution of interactions between plants and endophytes.

## Introduction

Evidence regarding the plant-associated microbial diversity in reproductive organs (i.e., the anthosphere, carposphere and spermosphere) is accumulating, which indicates the extensive existence of vertical transmission [1]. Microorganisms are ubiquitous across all environments [2], yet we are just beginning to understand their diversity and the role they play within ecosystems. Endophytes are microorganisms that live in the interstitial space of living cells of plant tissues in part or in all stages of their life cycle and do no obvious harm to plants [3]. According to the relationship between plants and bacteria, endophytic bacteria can be divided into obligate endophytic bacteria and facultative endophytic bacteria. Because most endophytes in plants come from the surrounding environment and rhizosphere soil, some weak pathogenic bacteria will colonize plants, occupy a certain niche and establish harmonious symbiotic relationships with host plants [4]. In addition to *Rhizobium*, other microbes such as *Bacillus*, *Xanthomonas*, *Pseudomonas*, *Erwinia*, *Pantoea*, *Burkholderia*, *Enterobacter* and *Agrobacterium* are all common microbes parasitizing varieties [5].

Several studies have demonstrated that the formation of bacterial assemblages in plants is not disorderly but rather bound by specific assembly rules [6]. The members of plant endophytes appear to emerge as a fundamental trait through diverse biochemical mechanisms to affect plant growth and development [7]. Moreover, the structural diversity of microorganisms is paramount in terms of the prevention of invasive pathogens/outbreaks [8]. Within plant-bacteria research, most functions of microbiota members that have been elucidated are beneficial toward plant health, including adapting to environmental variations [9], improving nutrient bioavailability, suppressing pathogens, eliciting plant immune systems, etc. On the other hand, the change in microbial community in soil, the increase in pathogenic microorganisms and the decrease in beneficial microorganisms are also one of the causes affecting crop growth [10].

Endophytes and hosts have formed a complex and special relationship due to long-term coexistence [11]. Some of them are mutually beneficial symbiotic relationships, while others are harmless or slightly harmful parasitic relationships. The two relationships can be transformed with various factors. The change in the community structure of endophytic bacteria in crops is an important aspect to reflect changes in the external environment [12]. It is necessary to study the genetic diversity of endophytic bacteria under different environmental factors to reveal their structure and function.

Chinese chive (*Allium tuberosum*) is a perennial herb of *Amaryllidaceous* widely cultivated in China. It is well known not only as a vegetable but also as a traditional medicinal material to treat nocturnal emissions, asthma, abdominal pain and diarrhoea [13]. In this study, three varieties of Chinese chive, Jiuxing 16, Jiuxing 18 and Jiuxing 23, were provided as host plants. High-throughput sequencing (Illumina-MiSeq) was employed to sequence the V5-V6 region of the 16S rRNA gene in bacteria from different samples. The sequence variation in the target region was detected, and the species richness and community structure of bacteria in the endosphere (inner tissues), ectosphere (outer surfaces), and the above- and below-ground compartments of Chinese chives were analysed to grasp the resource status of endophytic bacteria and to provide insight into the ecological function and interaction mechanisms with plants.

## Materials and methods

### Experimental design

The sampling site of the experiment was located in Henan Jiuxing Institute of Biotechnology, Pingdingshan, Henan Province, China (latitude, 33.34N; longitude, 113.03E), where three Chinese chive varieties, Jiuxing 16, Jiuxing 18 and Jiuxing 23, were planted for this test. Jiuxing 16, Jiuxing 18 and Jiuxing 23 are all appropriate varieties cultivated by Henan Jiuxing Institute of Biotechnology. The sampling work had been approved by Henan Jiuxing Institute of Biotechnology, which was the owner of the sampling site and the above-mentioned Chinese chive varieties. Furthermore, Henan Jiuxing Institute of Biotechnology was also one of the cooperative research institutions of this research project and the field studies did not involve endangered or protected species.

For the purpose of breeding and extension, their adaptability to extension needs to be studied continuously. Jiuxing 16 belongs to a non-dormant variety of Chinese chives that grow fast in spring and have dark green leaves. Jiuxing 18 is also a non-dormant variety, which has a strong cold resistance, strong growth potential and outstanding upright character. It has the characteristics of high resistance to grey mould and is the preferable variety planted in the late autumn. In winter, it can be planted in the open field in the south of Huang He River. The leaf colour of Jiuxing 18 is slightly lighter than that of Jiuxing 16, and its upright and cold resistance properties are better than that of Jiuxing 16. Jiuxing 23 is a new hybrid dormant variety that has high quality, disease resistance and high yield.

The rhizosphere is a very narrow area, not the soil taken directly from around the roots. Distinguished rhizosphere soil and non-rhizosphere soil according to the degree of shaking off and adhesion of soil on the surface of plant roots system, that is, the soil gently shaken off by man is regarded as non- rhizosphere soil, and the soil loosely adhered to the surface of root system within 1-4mm is rhizosphere soil [14].

In this experiment, the rhizosphere soil was strictly defined as above-mentioned soil particles adhering to the roots of Chinese chive and the samples of roots were collected at the depth of 5.0–20.0 cm below the ground level. A total of 45 samples from five different compartments (rhizosphere soil, root, stem, leaf and phylloplane) were collected from these three Chinese chive varieties on September 15, 2018. Corresponding to every variety of Chinese chives, there were triplicate samples that were collected from each compartment.

### Sample collection

For sample collection in the rhizosphere soil of Chinese chive, the plants were uprooted with an ethanol-sterilized spade [15]. The loosely adhering soil was shaken off carefully, and the tightly adhering soil was stripped by sterile swabs and collected frozen storage as rhizosphere soil sample. There were triplicate homogenized composite samples of rhizosphere soil from 12 randomly sites obtained corresponding to each variety aforementioned Chinese chives.

For sample collection on the phylloplane of Chinese chive, sterile swabs wet with sterilised water were used to swab each side of leaves one by one [16]. Triplicate phylloplane microbial samples from twelve randomly individuals were collected corresponding to each variety of aforementioned Chinese chives. Swab heads were cut off to fall into a sterile microcentrifuge tubes and stored frozen immediately until processing. At the same time, Triplicate samples in each plant tissue (leaf, stem and root) were also collected respectively using sterile scissors and stored frozenly in sterile polypropylene tubes.

### DNA extraction and amplicon selection

The pre-treatment procedure of cotton swabs is as follows: two cotton swabs were taken from each sample package, and the heads of cotton swab were put into 2.0 ml centrifuge tube. Then beads and cracking liquid were added in the centrifuge tube for the treatment of beating. For the pre-treatment of plant tissue samples, 0.2 g of plant tissue sample was put into 2.0 ml centrifuge tube in which liquid nitrogen was added. After the liquid nitrogen is completely volatilized, grind the plant tissue sample into powder, and add it into 1.0 ml cracking solution for mixing thoroughly.

Bacterial DNA was extracted from aforementioned five compartments using the FastDNA^®^SPIN Kit for Soil (Mpbio Bio-tek, USA) according to the manufacturer’s protocols. Furthermore, the final DNA concentration and purification were evaluated by a NanoDrop 2000 UV-vis spectrophotometer (Thermo Scientific, USA), and DNA quality was checked by 1% agarose gel electrophoresis (5 V, 20 min). The V5-V6 hypervariable regions of the bacterial 16S rRNA gene were amplified with the primers 799F (5’- AACMGGATTAGATACCCKG-3’) and 1193R (5’-ACGTCATCCCCACCTTCC-3’) according to the method mentioned in the reference [17].

The PCRs were conducted as follows: 3 min of initial denaturation at 95 °C, 27 cycles of 30 s at 95 °C, 30 s for annealing at 55 °C, and 45 s for elongation at 72 °C, and a final extension phase was completed at 72 °C for 10 min. The resulting PCR products were extracted from a 2% agarose gel, further purified using the AxyPrep DNA Gel Extraction Kit (Axygen Biosciences, USA) and quantified using QuantiFluor^™^-ST (Promega, USA) according to the manufacturer’s protocol.

Purified amplicons were pooled in equimolar and paired-end sequenced (2 × 300) on an Illumina MiSeq platform (Illumina, San Diego, USA) according to the standard protocols by Majorbio Bio-Pharm Technology Co. Ltd (Shanghai, China).

### Sequence processing and OTU designation

The raw reads were deposited into the database of National Center for Biotechnology Information (NCBI) Sequence Read Archive (SRA). Raw fastq files were de-multiplexed, quality-filtered by Trimmomatic [18] and merged by FLASH (fast length adjustment of short reads) [19]. Operational taxonomic units (OTUs) were clustered with a 97% similarity cutoff by UPARSE, which is a method for generating clusters (OTUs) from next-generation sequencing reads of marker genes (version 7.1 http://drive5.com/uparse/), and chimeric sequences were identified and removed by UCHIME (algorithms for detecting chimeric sequences) [20]. The taxonomy of each 16S rRNA gene sequence was analysed by the RDP Classifier algorithm (http://rdp.cme.msu.edu/) against the SILVA (Release 132) 16S rRNA database using a confidence threshold of 70%.

### Statistical analysis

Before estimating differences in the alpha diversity, the singletons were removed (OTUs with only one sequence) from the dataset since these singletons could be due to sequencing artefacts. Rarefaction curves showing the number of OTUs defined at the 97% sequence similarity cut-off relative to the number of total sequences were assembled in Mothur. To determine the significance of Chinese chive compartments as grouping factors, the principal coordinate analysis (PCoA) and adonis analyses of each sample was carried out in Mothur. Pie graph was produced in R project. To demonstrate the composition of identified community members within different plant compartments at the phylum level. Statistical analysis was performed by one-way ANOVA. P values were corrected for multiple comparisons using the false discovery rate (FDR) with the Benjamini–Hochberg method. To investigate the representative genera of microorganisms in different compartments of Chinese chives, the relative richness of microbes in each compartment was determined by the random forest machine learning algorithm [21]. The number of decision trees was set to 500, and the 10-fold cross validation method was adopted to calculate the importance of each bacterial genus in these different compartments of Chinese chives. A heatmap was produced in R project with the “heatmap” package.

## Result

### Validation of the sequencing data accuracy

Across the 45 samples collected from different species of Chinese chives, the community structure of endophytic bacteria in the five compartments was characterized by Illumina MiSeq sequencing of the bacterial V5-V6 region of the 16S rRNA gene. A total of 2762429 high-quality reads with an average length of 394.41 bp ± 0.82 were obtained after denoising (Table 1). The estimated sample coverage (Good’s coverage) in each group was more than 98%, which indicated that the accuracy of sequencing was reliable.

**Table 1:** Summary of sequencing data and community diversity.

	Rhizosphere soil	Root endosphere	Stem endosphere	Leaf endosphere	Phylloplane
Sequences	46042±13539	51222±12732	42288±9152	47455±13546	43196±11066
Average length	394.70±0.76	395.52±1.01	394.03±0.83	393.93±0.85	393.88±0.65
OTUs	895.78±168.19	499.22±126.49	274.56±30.72	260.00±47.85	353.56±87.27
Good’s coverage	0.984±0.003	0.989±0.003	0.996±0.001	0.996±0.001	0.993±0.002
Chao1	1169.50±200.44	712.96±178.72	331.57±46.66	345.93±50.37	482.33±143.4
Shannon	5.10±0.51	3.48±0.79	3.48±0.15	3.48±0.30	3.81±0.22

Data represent the mean ± standard deviation (S.D.). The number of OTUs, Good’s coverage scores, richness estimator Chao, and diversity estimator Shannon were calculated at 3% distance. n = 12 in each group. OTU, operational taxonomic unit.

**Fig 1:**
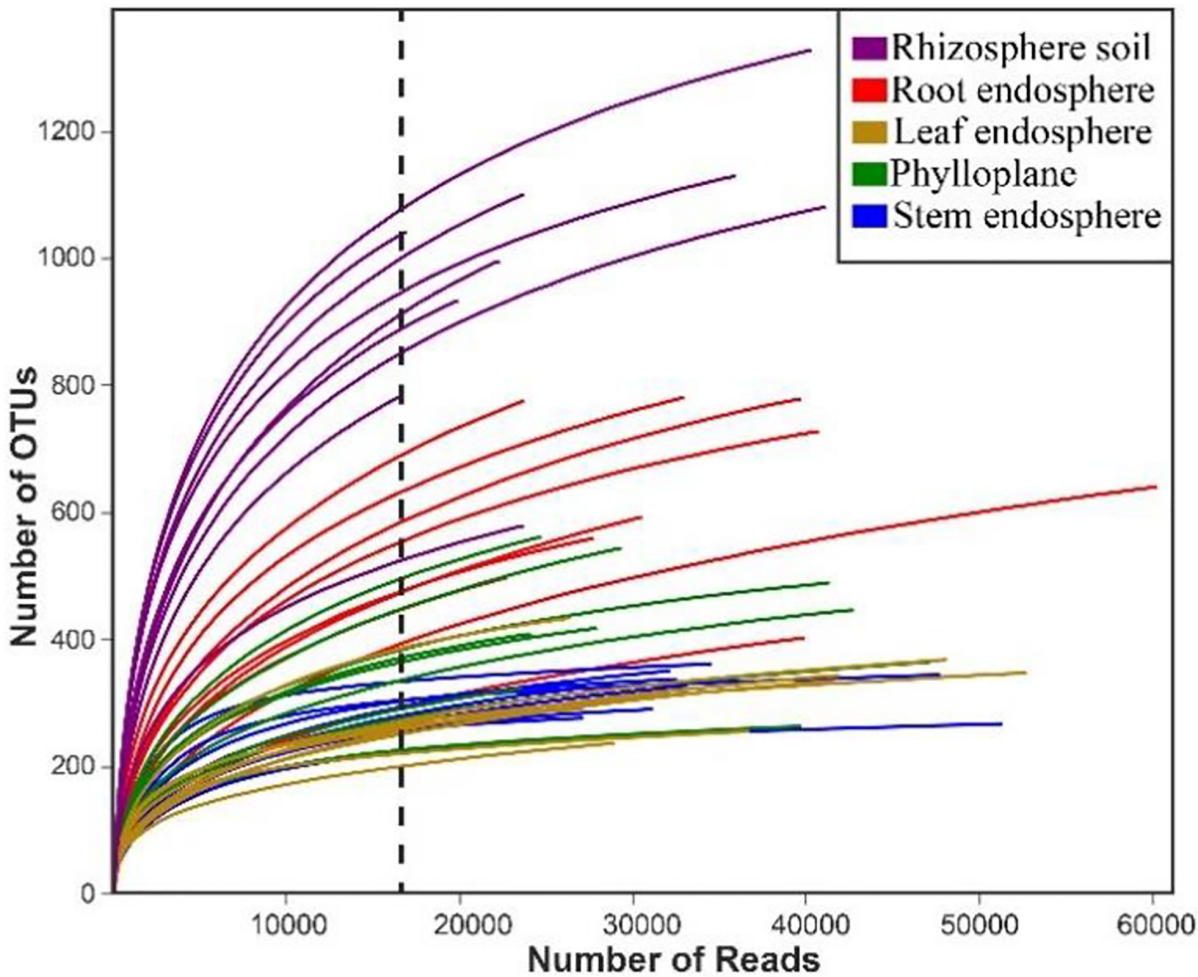
Rarefaction curves of each Chinese chive compartment.

Rarefaction curves showing the number of OTUs (Fig 1). The dashed vertical line indicates the numbers of sequences subsampled from each sample. In addition, the gradual flattening of the rarefaction curves indicates that the sequencing quantity of each sample is close to saturation, which is consistent with the results obtained by the Good's coverage index (Table 1).

**Fig 2:**
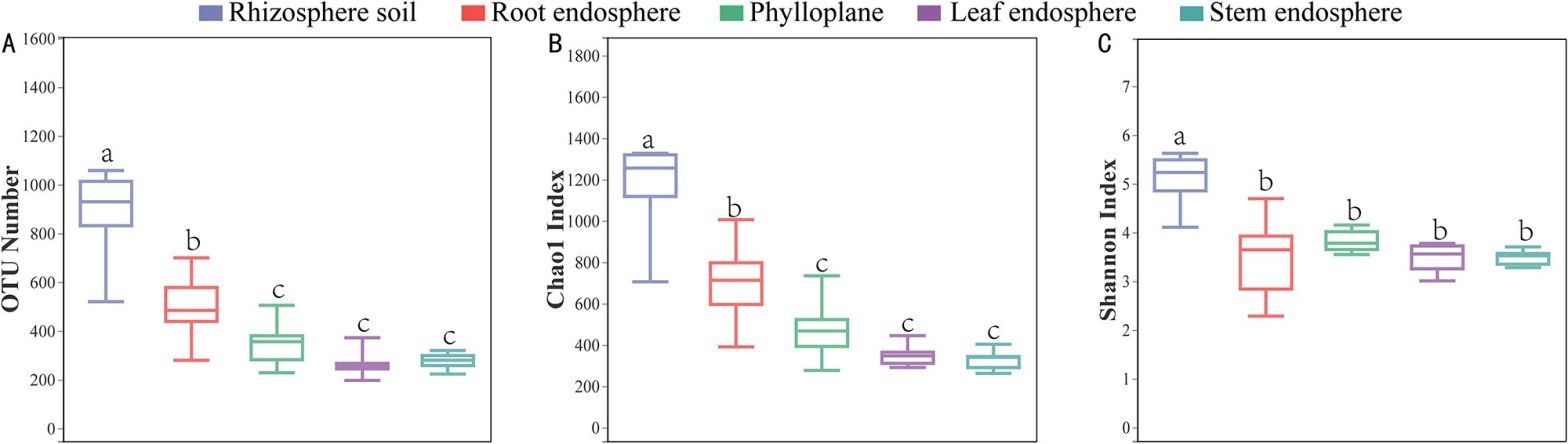
Alpha diversity indices of the 16S rRNA gene sequences. Box plots of the observed OTUs, Chao1 indices, and Shannon indices in the rhizosphere soil, root endosphere, stem endosphere, leaf endosphere and phylloplane. Whiskers represent the minimum and maximum values, and the bar represents the median. Data were analysed by means of Student’s t test, and significant differences (P < 0.05) across plant compartments are indicated with lowercase letters.

From the construction of the alpha rarefaction curve, it can be seen that the number of OTUs in the above-ground samples was far less than that in the rhizosphere samples (Fig 2). Higher species richness of microbes in the group of rhizosphere soil exhibited significantly higher OTU numbers (P < 0.05), Chao1 indices (P <0.05), and Shannon (P <0.05) indices compared with those of the rest groups (Fig 2).

#### Overall structural changes in the different compartments

**Fig 3:**
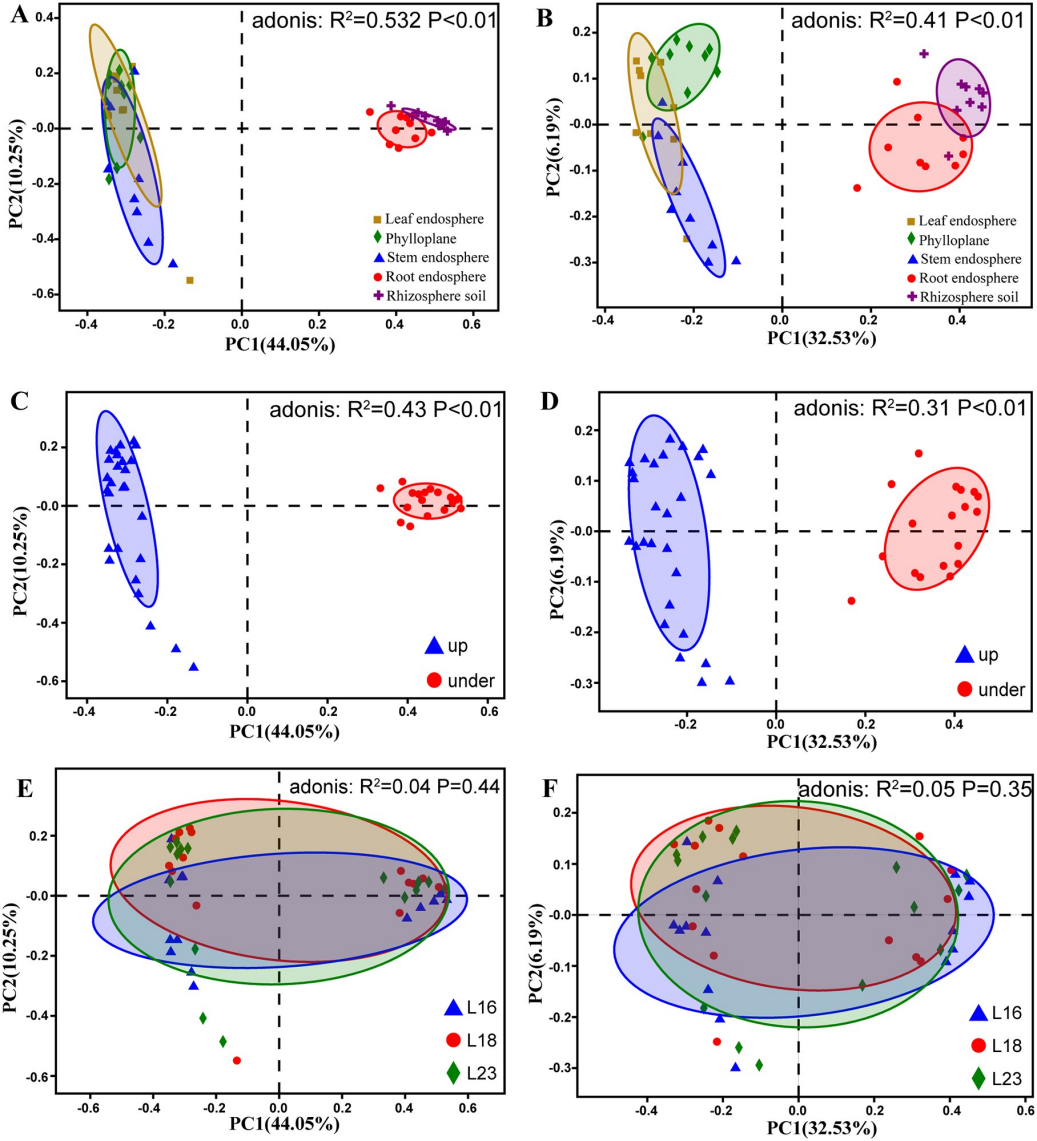
Bacteria are separable by plant compartments at the OTU level. (A) PCoA using the Bray-Curtis method coloured to depict the plant compartments. (B) PCoA using the Binary-Jaccard method coloured to depict the plant compartments. (C) PCoA using the Bray-Curtis method coloured to depict the vertical stratification. (D) PCoA using the Binary-Jaccard method coloured to depict the vertical stratification. (E) PCoA using the Bray-Curtis method coloured to depict the species. (F) PCoA using the Binary-Jaccard method coloured to depict the species. Statistical support for the PCoA clustering is provided by the permutational multivariate analysis of variance (adonis).

PCoA and adonis analyses showed that bacterial communities in each Chinese chive compartments were significantly different as grouping factors (p < 0.01) (Fig 3).

When plant compartments were used as grouping factors (Fig 3A and 3B), rhizosphere soil and root endosphere groups clustered on the right side of the PCoA graph, and stem endosphere, leaf endosphere and phylloplane groups clustered on the left side of the PCoA graph. Adonis analysis showed that when the grouping factors were measured by the Bray-Curtis distance algorithm (R^2^ = 0.532, P < 0.01) and the Binary-Jaccard distance algorithm (R^2^ = 0.41, P < 0.01), there were significant differences in the results of grouping according to plant compartments.

When the vertical stratification structure of plants was taken as the grouping factor (Fig 3C and 3D), the above-ground group gathered on the left side of the PCoA graph, and the below-ground group gathered on the right side of the PCoA graph. Adonis analysis showed that when it was measured by the Bray-Curtis distance algorithm (R^2^ = 0.43, P < 0.01) and the Binary-Jaccard distance algorithm (R^2^ = 0.31, P < 0.01), the vertical stratification structure of plants as the grouping factor could lead to significant differences in the calculation results.

PCoA indicated that there was no obvious visual clustering when the varieties of Chinese Chive were used as the grouping factor (Fig 3E and 3F). Adonis analysis based on the Bray-Curtis distance algorithm (R^2^ = 0.04, P = 0.44) and Binary-Jaccard distance algorithm (R^2^ = 0.05, P = 0.35) also showed that there were no significant differences in the calculated results.

#### Bacterial community composition in different compartments

**Fig 4:**
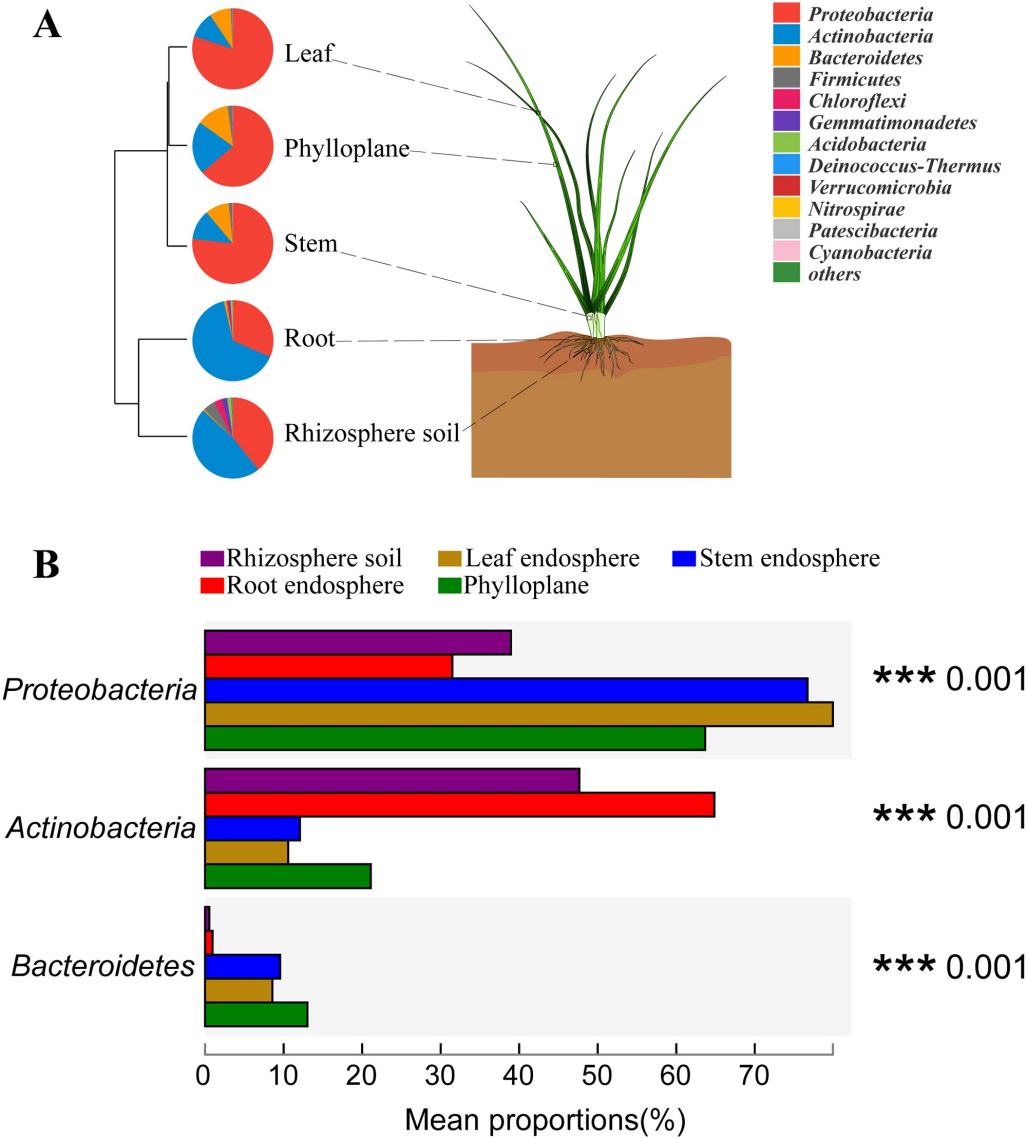
Bacterial community at the phylum level (A) and statistical comparison of the top three phyla (B). The major contributing phyla across niches within the habitats of rhizosphere soil, root endosphere, stem endosphere, leaf endosphere and phylloplane were displayed in different colours. n = 12, in each group. *P < 0.05, **P < 0.001.

To demonstrate the composition of identified community members within different plant compartments at the phylum level, the microbial composition of each group was depicted by a pie graph (Fig 4A). At the phylum level, the three most abundant microbes in the five compartments were *Proteobacteria*, *Actinobacteria*, and *Bacteroidetes*, and they represented 87.15–99.09% of the 16S rRNA gene sequences.

*Proteobacteria* with the highest richness in the above-ground compartments and *Actinobacteria* with the highest richness in the below-ground compartments indicated that the composition and structure of bacteria at the phylum level in both above-ground compartments and below-ground compartments were strikingly different (Fig 4B). Most of the identified bacteria showed significant differences at the phylum level except *Patescibacteria* (P = 0.068) and *Chlamydiae* (P = 0.065) (Table 2). This means that different compartments significantly affected the composition of bacteria.

**Table 2:** Effect of plant compartments on the individual bacterial phyla.

	Mean ± Sd (%)					P-value
Phylum	Rhizosphere soil	Root endosphere	Stem endosphere	Leaf endosphere	Phylloplane	
*Proteobacteria*	38.95±8.62	31.49±18.49	76.71±8.64	79.94±6.82	63.68±9.59	<0.0001
*Actinobacteria*	47.66±8.84	64.85±20.31	12.08±6.03	10.58±3.18	21.1±7.74	<0.0001
*Bacteroidetes*	0.54±0.46	0.96±2.19	9.56±7.96	8.57±5.29	13.03±5.07	<0.0001
*Firmicutes*	4.872±2.711	0.297±0.273	1.311±1.269	0.313±0.295	1.988±2.325	0.0007
*Chloroflexi*	3.119±1.033	0.867±0.533	0.030±0.036	0.014±0.020	0.012±0.027	<0.0001
*Gemmatimonadetes*	2.231±1.314	0.464±0.429	0.021±0.020	0.008±0.020	0.003±0.018	0.0004
*Acidobacteria*	1.603±0.774	0.836±0.624	0.047±0.054	0.011±0.020	0.017±0.017	<0.0001
*Deinococcus-Thermus*	0	0	0.044±0.046	0.429±0.711	0.076±0.078	NA
*Verrucomicrobia*	0.306±0.127	0.053±0.054	0.005±0.014	0	0.001±0.002	NA
*Nitrospirae*	0.267±0.172	0.014±0.010	0.007±0.007	0.002±0.004	0.002±0.004	0.0009
*Patescibacteria*	0.123±0.130	0.103±0.186	0.003±0.001	0.001±0.002	0.001±0.003	0.0676
*Armatimonadetes*	0.047±0.035	0.014±0.018	0.006±0.006	0.079±0.011	0.031±0.041	0.0301
*Cyanobacteria*	0.031±0.030	0.005±0.006	0.120±0.043	0.018±0.004	0.003±0.005	0.0351
*Planctomycetes*	0.084±0.056	0.011±0.012	0.014±0.013	0.002±0.004	0.003±0.004	0.0036
*Chlamydiae*	0.059±0.056	0.008±0.012	0.003±0.006	0.005±0.012	0.002±0.003	0.0648
*FBP*	0.009±0.016	0.002±0.003	0.001±0	0.023±0.027	0.034±0.036	0.0189
*Dependentiae*	0.045±0.043	0.011±0.014	0	0	0.001±0.003	NA
*Fusobacteria*	0	0	0.048±0.137	0	0	NA
*Elusimicrobia*	0.028±0.023	0.005±0.005	0	0	0	NA
*unclassified_k__norank*	0.005±0.008	0.008±0.014	0	0.001±0.003	0.001±0.004	NA
*Latescibacteria*	0.015±0.038	0±0	0	0	0	NA
*Fibrobacteres*	0.003±0.007	0.001±0.002	0.001±0.002	0	0	NA
*Entotheonellaeota*	0.001±0	0.001±0.004	0	0	0.001±0.004	NA
*Deferribacteres*	0.003±0.004	0	0	0	0	NA
*Kiritimatiellaeota*	0.001±0.004	0	0	0	0	NA
*Spirochaetes*	0	0	0.001±0.002	0	0	NA

Significant differences in the variance of parameters were evaluated with ANOVA.

**Fig 5:**
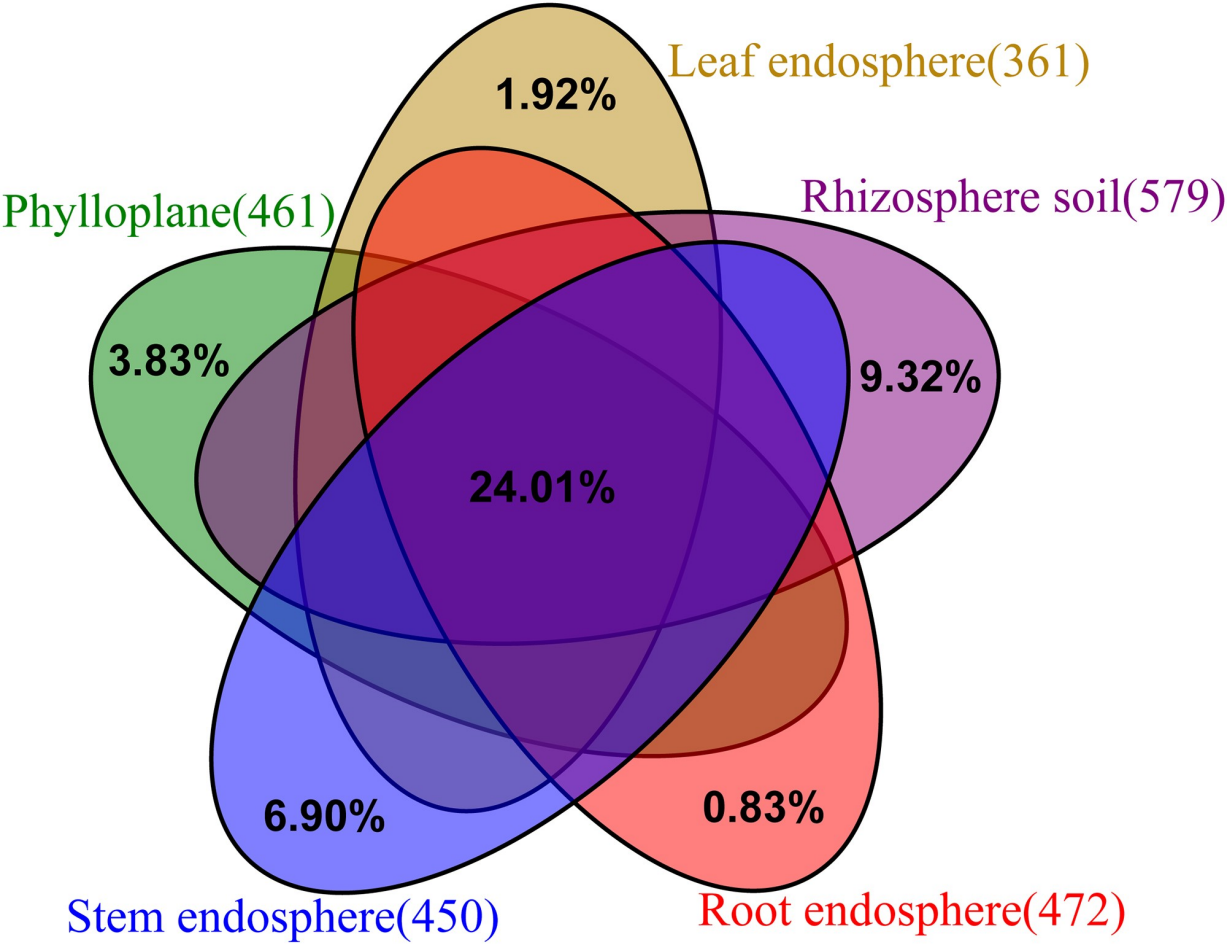
Unique and shared genera presented in the different compartments.

Next, the changes in microbial structure from different compartments at the genus level were analysed, and 783 genera were identified. The rhizosphere soil contained 579 genera, the leaf endosphere contained 361 genera, and 183 genera were jointly owned by the five compartments, which accounted for 24.01% (Fig 5). The ten most abundant genera in these compartments are listed separately in S1 Table.

#### Biomarkers in different compartments

**Fig 6:**
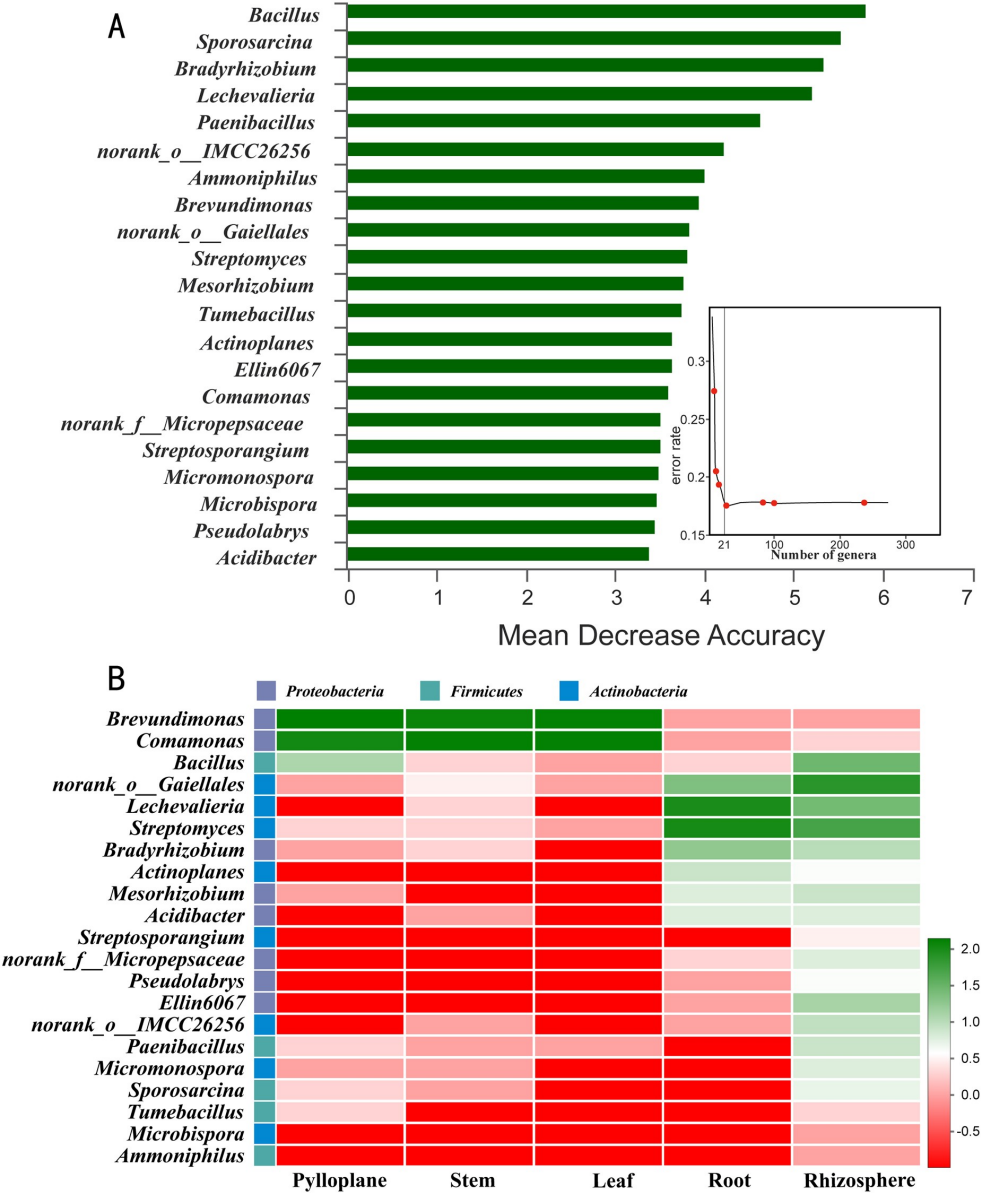
Bacterial taxonomic biomarkers across niches within the stem, phylloplane, leaf, root and rhizosphere. (A) The top 21 biomarker bacterial classes were identified by applying random forest regression of their relative richness. Biomarker taxa are ranked in descending order of importance to the accuracy of the model. The insert represents a 10-fold cross-validation error as a function of the number of input classes used to regress against the different plant compartments in order of variable importance. (B) Heatmap showing the relative richness of the top 21 biomarker bacterial classes against the different plant compartments.

Random forest machine learning algorithm was used to investigate the representative genera of microorganisms in different compartments of Chinese chives. The results indicated that 21 bacterial genera could be used as biomarkers (Fig 6A), and the relative richness of these biomarkers in different compartments of Chinese chive are shown in Fig 6B. Furthermore, *Brevundimonas* and *Comamonas* had higher richness in the leaves, phylloplanes and stems in this experiment. The proportions of *Streptomyces*, *Lechevalieria*, *Actinoplanes*, *Bradyrhizobiaceae*, *norank_o_Gaiellales*, and *Mesorhizobium* in the root endosphere and rhizosphere were relatively high.

## Discussion

Endophytes are widely distributed in almost all known plants, mainly in the tissues or intercellular spaces of roots, stems, leaves, flowers and fruits. The species, quantity and distribution of endophytes vary with plant species. Soil bearing endophytes are thought to initially infect host plants through abrasions caused by microbial or nematode phytopathogens from where they can quickly spread to intercellular spaces in the roots [22]. In this study, we found that the OTU numbers in the rhizosphere compartment were higher than those in the other compartments of Chinese chives (Table 1, Figs 1 and 2). Clearly, the microbial species richness in the rhizosphere was higher than that of root-endosphere [23–25]. This is related to rhizodeposition and root exudation of host plants, which can fuel chemoattraction and colonization of the rhizosphere soil and rhizoplane, resulting to the formation of distinctive, highly rich, and diverse rhizosphere microbiomes [26–29].

Despite the large number of bacterial phyla described in nature and the multiple factors that affect these communities, the bacterial microbiota of plants is dominated by three major phyla (*Proteobacteria*, *Actinobacteria*, *and Bacteroidetes*) in both the above- and below-ground plant tissues [30,31]. This is also consistent with our research (Fig 4). We found that at the level of phylum, *Proteobacteria* is the most abundant in the above-ground compartments, and *Actinobacteria* is the most abundant in the below-ground compartments.

A considerable number of endophytic bacteria form symbiotic and mutually beneficial relationships with host plants, which play an important role in regulating host growth, metabolism and stress resistance. It has been proved that *Methylobacterium* was abundantly present in both leaf endosphere and phylloplane [32], showed specific colonization strategies by profiting from methanol that is released by the plant as a by-product [33]. Furthermore, enrichment of *Streptomyces* and *Burkholderia-Caballeronia-Paraburkholderia* in both root endosphere and rhizosphere soil were linked to pathogen suppression [34]. In addition, some researchers reported that the community structures of the microbiome were significantly different among different plant species [35]. The richness of endophytic bacteria also varies greatly among different varieties of Chinese chive. For example, at the genera level, *Lechevalieria*, *norank_o__Gaiellales*, and *Burkholderia-Caballeronia-Paraburkholderia* had the highest richness in the rhizosphere of Jiuxing 16, Jiuxing 18 and Jiuxing 23, respectively, and *Lechevalieria*, *Lechevalieria* and *Streptomyces* had the highest richness in the root endosphere of Jiuxing 16, Jiuxing 18 and Jiuxing 23, respectively.

The bacteria that colonize plants and can fix nitrogen are called endophytic diazotroph. Compared with the rhizosphere Azotobacter, the endophytic diazotroph is more favorable to utilize the carbon source provided by the host plant, and the relatively low oxygen environment in the host plant is also conducive to the expression and function of microbial nitrogenase. In this study, *Bradyrhizobium*, *Burkholderia* and *Pseudomonas* were found possessing some characteristics of endophytic diazotroph, and their effect of promoting the growth of Chinese chives still need further study.

## Conclusion

Endophytes mainly promote the growth and development of host plants by producing plant hormones, dissolving phosphorus, potassium and other nutrients in soil, antagonizing plant pathogenic microorganisms and producing resistance to biotic or abiotic stresses. For the first time, high-throughput sequencing technology was used to differentiate the species richness of bacterial communities between the endosphere (inner tissues) and ectosphere (outer surfaces) of Chinese chives. Alpha diversity index analysis and OTU number analysis showed that the bacterial diversity and richness of the below-ground plant compartments were higher than those of the above-ground parts.

PCoA based on the OTU level showed that the vertical stratification structure of plants and compartments had significant effects on bacterial community structure. This indicated that each plant compartment represents a unique niche of bacterial communities, and endophytic bacteria are selective and tissue-specific in plants. At the same time, 21 bacterial genera were identified as biomarkers in the different compartments. The determination of biomarkers has laid the foundation for further understanding of the microbial interactions in different compartments of Chinese chives.

## Supporting information

S1 Table Top 10 genera in the different compartments. (DOCX)
