# Supplementary material for: The variability of bacterial communities in both the endosphere and ectosphere of different niches in Chinese chives (Allium tuberosum)
Source: PLoS One. 2020 Jan 16;15(1):e0227671. doi: 10.1371/journal.pone.0227671 (PMC6964972; doi:10.1371/journal.pone.0227671)
Supplement: S1 Table — (DOCX) [file pone.0227671.s001.docx]

**S1 Table. Top 10 genera in the different compartments.**

| **Plant compartment** | **Genus** | **Relative abundance (%)** | | | |
| --- | --- | --- | --- | --- | --- |
|  |  | **Jiuxin16L** | **Jiuxing18L** | **jiuxing23L** | **Mean** |
| Root endosphere | *Lechevalieria* | 29.45 | 22.20 | 3.39 | 18.35 |
|  | *Streptomyces* | 13.42 | 2.94 | 13.61 | 9.99 |
|  | *Pseudoduganella* | 10.18 | 0.52 | 2.00 | 4.23 |
|  | *unclassified_f__Burkholderiaceae* | 2.46 | 0.69 | 4.05 | 2.40 |
|  | *Marmoricola* | 2.44 | 0.46 | 1.34 | 1.41 |
|  | *Bradyrhizobium* | 2.15 | 1.93 | 1.36 | 1.81 |
|  | *Actinocorallia* | 1.93 | 0.00 | 0.80 | 0.91 |
|  | *Phycicoccus* | 1.90 | 0.27 | 1.07 | 1.08 |
|  | *unclassified_f__Micromonosporaceae* | 1.77 | 0.12 | 0.74 | 0.88 |
|  | *Burkholderia-Caballeronia-Paraburkholderia* | 1.62 | 1.79 | 17.53 | 6.98 |
|  | **Total** | **67.31** | **30.91** | **45.87** | **48.03** |
| Rhizosphere soil | *Burkholderia-Caballeronia-Paraburkholderia* | 3.06 | 5.96 | 22.50 | 10.51 |
|  | *norank_o__Gaiellales* | 8.08 | 7.19 | 8.71 | 7.99 |
|  | *Streptomyces* | 6.17 | 5.01 | 4.84 | 5.34 |
|  | *Lechevalieria* | 9.71 | 1.01 | 0.25 | 3.65 |
|  | *norank_f__67-14* | 1.52 | 4.15 | 2.14 | 2.61 |
|  | *Bacillus* | 3.05 | 1.22 | 3.18 | 2.48 |
|  | *Sphingomonas* | 1.69 | 3.76 | 1.75 | 2.40 |
|  | *norank_f__JG30-KF-AS9* | 2.19 | 2.23 | 2.48 | 2.30 |
|  | *Conexibacter* | 1.76 | 2.65 | 1.74 | 2.05 |
|  | *Jatrophihabitans* | 1.41 | 2.25 | 2.26 | 1.97 |
|  | **Total** | **38.65** | **35.42** | **49.85** | **41.31** |
| Leaf endosphere | *Sphingomonas* | 20.28 | 23.26 | 37.31 | 26.95 |
|  | *Methylobacterium* | 24.57 | 7.70 | 22.08 | 18.12 |
|  | *Curtobacterium* | 8.18 | 4.64 | 4.61 | 5.81 |
|  | *Pantoea* | 10.97 | 4.10 | 0.72 | 5.26 |
|  | *Chryseobacterium* | 5.54 | 4.32 | 3.98 | 4.62 |
|  | *Massilia* | 4.87 | 5.94 | 1.02 | 3.94 |
|  | *Brevundimonas* | 2.45 | 5.75 | 3.15 | 3.78 |
|  | *Comamonas* | 1.85 | 7.12 | 2.08 | 3.69 |
|  | *Allorhizobium-Neorhizobium-Pararhizobium-Rhizobium* | 2.89 | 4.66 | 3.43 | 3.66 |
|  | *Microbacterium* | 3.19 | 1.08 | 4.05 | 2.77 |
|  | **Total** | **84.79** | **68.56** | **82.43** | **78.59** |
| Phylloplane | *Sphingomonas* | 20.95 | 20.59 | 21.82 | 21.12 |
|  | *Curtobacterium* | 12.00 | 7.70 | 18.41 | 12.70 |
|  | *Chryseobacterium* | 8.10 | 9.46 | 13.80 | 10.45 |
|  | *Massilia* | 7.93 | 12.96 | 2.17 | 7.69 |
|  | *Methylobacterium* | 7.39 | 4.74 | 10.47 | 7.53 |
|  | *Pantoea* | 4.98 | 5.31 | 3.48 | 4.59 |
|  | *Pseudomonas* | 4.58 | 4.33 | 1.78 | 3.57 |
|  | *Brevundimonas* | 5.07 | 3.45 | 1.97 | 3.50 |
|  | *Stenotrophomonas* | 1.87 | 4.46 | 3.40 | 3.24 |
|  | *Microbacterium* | 2.80 | 3.80 | 2.92 | 3.18 |
|  | **Total** | **75.68** | **76.79** | **80.22** | **77.56** |
| Stem endosphere | *Sphingomonas* | 26.96 | 32.20 | 21.82 | 26.99 |
|  | *Methylobacterium* | 8.28 | 15.95 | 10.47 | 11.57 |
|  | *Curtobacterium* | 6.69 | 8.48 | 18.41 | 11.19 |
|  | *Chryseobacterium* | 0.37 | 1.28 | 13.80 | 5.15 |
|  | *Pseudomonas* | 9.78 | 3.29 | 1.78 | 4.95 |
|  | *Pantoea* | 3.78 | 6.30 | 3.48 | 4.52 |
|  | *Brevundimonas* | 6.00 | 5.17 | 1.97 | 4.38 |
|  | *Comamonas* | 7.97 | 1.32 | 0.85 | 3.38 |
|  | *Allorhizobium-Neorhizobium-Pararhizobium-Rhizobium* | 2.92 | 3.90 | 1.53 | 2.78 |
|  | *Stenotrophomonas* | 0.87 | 3.10 | 3.40 | 2.46 |
|  | **Total** | **73.61** | **80.99** | **77.52** | **77.37** |
